# Thelaziosis in Humans, a Zoonotic Infection, Spain, 2011

**DOI:** 10.3201/eid1812.120472

**Published:** 2012-12

**Authors:** Isabel Fuentes, Isaías Montes, Jose M. Saugar, Stefania Latrofa, Teresa Gárate, Domenico Otranto

**Affiliations:** Author affiliations: Instituto de Salud Carlos III, Madrid, Spain (I. Fuentes, J.M. Saugar, T. Gárate);; Hospital Ciudad de Coria, Cáceres, Spain (I. Montes);; Università degli Studi di Bari, Valenzano, Italy (S. Latrofa, D. Otranto)

**Keywords:** Thelazia callipaeda, parasites, helminths, thelaziosis, humans, eye, zoonosis, diagnosis, zoonotic infection, Phortica variegate, Spain

## Abstract

After *Thelazia callipaeda* infection in dogs and cats were reported in Spain, a human case of thelaziosis in this country was reported, suggesting zoonotic transmission. The active reproductive status of this nematode in situ indicates that humans are competent hosts for this parasite.

*Thelazia callipaeda* (Spirurida, Thelaziidae) is a parasitic helminth transmitted by zoophilic insects of the order Diptera, family Drosophilidae, genus *Phortica* while feeding on ocular secretions of their hosts during summer (*1**,**2*). The parasitic first-stage larvae are ingested by the vectors along with the conjunctival secretions of infected animals; they mature into their third larval stage in 2–3 weeks; and they are released as third-stage infective larvae into the eye of a new host (*2*). Nematodes localize in the orbital cavity and associated tissues of canids, felids, rodents, and humans, causing mild (i.e., lacrimation, itching, exudative conjunctivitis) to severe (i.e., corneal ulceration and keratitis) signs and symptoms, if not properly treated (*3**,**4*).

*T. callipaeda* has long been called the oriental eyeworm, referring to its traditional distribution across eastern and southeastern Asia (i.e., China, North and South Korea, Japan, Indonesia, Thailand, and India) where infection is endemic in animals and humans (*5*), usually in poorer rural areas and mainly among children and the elderly. Since the first cases of canine thelaziosis identified in Europe, which were in northern Italy in 1988 (*6*), several studies have indicated that the disease is endemic throughout Italy (*7*). In recent years, thelaziosis in cats and dogs has also been reported in France, Germany, and Switzerland, highlighting the spread of the disease in Europe (*8*). Autochthonous cases of *T. callipaeda* infection among dogs have recently been reported in Spain near the western part of the country (La Vera, Cáceres); prevalence in some municipalities has reached 39.9% of dogs examined (*9*).

After the parasite spread among domestic and wild carnivores from Europe, the first human cases of this zoonotic disease in Italy and France were described (*10*). Although humans are competent hosts, they usually act as accidental-ending hosts in whom the third stage larvae can grow into adults but without epidemiologic effects on parasite transmission. This lacking of effect could be explained because humans, in contrast to animals, are likely to report symptoms and consequently have parasites removed, causing the interruption of transmission.

We report a case of human thelaziosis in Spain. This report highlights the emerging nature of this zoonotic disease and calls for attention to its possible public health consequences. In addition, the finding of a mature female parasite with developed larvae in the uterus suggests that humans may be proper hosts for *T. callipaeda* development in areas where thelaziosis is endemic in dogs or cats.

## The Study

An adolescent girl, 17 years of age, from the village of Coria in the Province of Cáceres, Spain (40°N, 6°32′W), sought assistance at the regional hospital (Ciudad de Coria Hospital) in September 2011, describing the sensation of a foreign body in her left eye for 3 weeks. She reported having spent her holidays in the Cáceres countryside during July and August. Examination revealed lacrimation and conjunctival abnormalities or exudate; ophthalmologic examination revealed 2 filiform worms on the conjunctival fornix of the affected eye. Physical examination and laboratory analysis of blood showed results otherwise within reference values. After extraction of the slender worms with forceps, her symptoms resolved; an ophthalmic examination performed 2 weeks later yielded results within reference range.

The smaller of the 2 worms could not be retrieved for examination because it was destroyed during extraction, whereas the larger one was placed in physiologic saline solution and sent to the Centro Nacional de Microbiología, where it was fixed in 70% ethanol and identified on the basis of morphologic keys (*11*).

The retrieved worm was an adult female (17 mm long and 424 µm wide) and had a serrated cuticle with transverse striations (290/mm in the cephalic region, 200/mm in the midsection, and 240/mm in the tail section). The buccal capsule (28 µm wide, 26 µm deep) showed a hexagonal profile and 6 festoons (Figure). The vulva was anterior to the esophageal–intestinal junction; embryos were visible in the proximal area of the uterus, whereas larvae were visible in the distal area. The small size of the other worm (not recovered) and the active reproductive status of the female carrying young larvae suggested the unrecovered worm was most likely a male, who mated with the female in the patient’s orbital socket. 

**Figure:**
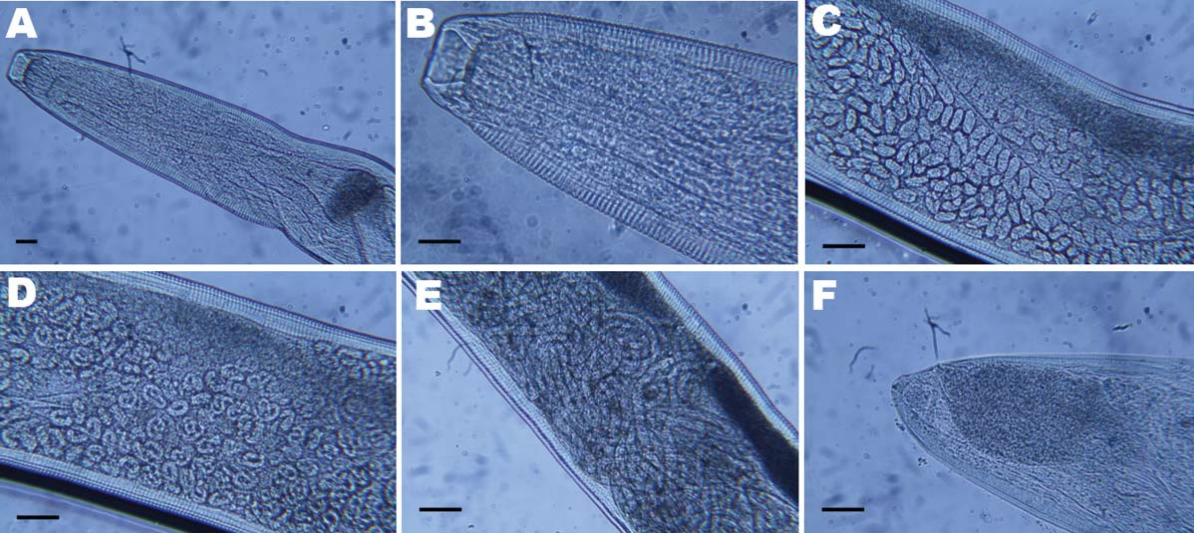
Light micrographs of *Thelazia callipaeda* showing A) posterior and B) anterior portion with cephalic end and buccal capsule; C) anterior portion containing embryonated eggs; D) middle portion containing rounded first-stage larvae; E) posterior portion containing first-stage larvae; F) caudal end. Scale bars = 25 µm.

The morphologic characteristics of the female worm led to its identification as *T. callipaeda*, which was molecularly confirmed by a specific PCR amplification (*12*). In brief, after we extracted genomic DNA from the female worm with the QIAamp DNA Mini Kit (QIAGEN, Hilden, Germany), a partial sequence of the mitochondrial cytochrome *c* oxidase subunit 1 gene (*cox*1, 689 bp) was amplified by PCR. Amplicons were purified by using Amicon Ultrafree-DA columns (Millipore, Bedford, MA, USA) and sequenced in an ABI-PRISM 377 by using the *Taq* DyeDeoxyTerminator Cycle Sequencing Kit (Applied Biosystems, Monza, Milan, Italy). Sequences were determined in both directions and aligned by using the ClustalX program (www.clustal.org). The alignments were verified and compared with the sequences available for the *cox*1 of *T. callipaeda* (GenBank accession nos. AM042549–556). Sequences obtained from the nematode were identical to the sequence representing haplotype 1 of *T. callipaeda* (GenBank accession no. AM042549) (*12*).

## Conclusions

We described a case of human thelaziosis in Spain in a patient living in a geographic region where 182 (39.9%) of the 456 dogs examined were recently found positive for *T. callipaeda* by morphologic and molecular analyses (*9*). These data indicate that in an area where thelaziosis is endemic in animal populations, there is also a risk for parasitization in humans. According to what is known of the biology of *T. callipaeda* in dogs and vectors in Europe (*1**,**2*), the end of summer is the period of maximum activity of the vector (*1*). Summer is when the human patient reported ocular discomfort. The patient had signs and symptoms of a mild infection including eye discomfort and foreign body sensation; a proper diagnosis was made after 3 weeks.

Considering the high prevalence of infection in dogs reported in recent studies and the case of human thelaziosis here described, general physicians and ophthalmologists should take human thelaziosis into account in their differential diagnoses of conjunctivitis, ocular lacrimation, and corneal ulcers (*4*). Medical continuing education and awareness of this condition are needed to ensure that the infection does not go undiagnosed and that appropriate treatment for the primary problem and for complications such as allergic reaction or bacterial infection can be prescribed.
